# Whole-Genome Sequencing Reveals Recent Transmission of Multidrug-Resistant *Mycobacterium tuberculosis* CAS1-Kili Strains in Lusaka, Zambia

**DOI:** 10.3390/antibiotics11010029

**Published:** 2021-12-28

**Authors:** Joseph Yamweka Chizimu, Eddie Samuneti Solo, Precious Bwalya, Wimonrat Tanomsridachchai, Herman Chambaro, Misheck Shawa, Thoko Flav Kapalamula, Patrick Lungu, Yukari Fukushima, Victor Mukonka, Jeewan Thapa, Chie Nakajima, Yasuhiko Suzuki

**Affiliations:** 1 Division of Bioresources, International Institute for Zoonosis Control, Hokkaido University, Kita 20 Nishi 10, Kita-ku, Sapporo 001-0020, Japan; chizimu@czc.hokudai.ac.jp (J.Y.C.); preciousbwalya@czc.hokudai.ac.jp (P.B.); wimonrat@czc.hokudai.ac.jp (W.T.); thokokapalamula@czc.hokudai.ac.jp (T.F.K.); yukaring@czc.hokudai.ac.jp (Y.F.); jeewan@czc.hokudai.ac.jp (J.T.); 2 Zambia National Public Health Institute, Ministry of Health, Lusaka 10101, Zambia; victor.mukonka@znphi.co.zm; 3 University Teaching Hospital, Ministry of Health, Lusaka 10101, Zambia; esolo@uthlabs.gov.zm; 4 Department of National Parks and Wildlife, Ministry of Fisheries and Livestock, Lusaka 10101, Zambia; herman@czc.hokudai.ac.jp; 5 Division of Molecular Pathobiology, Hokkaido University International Institute for Zoonosis Control, Sapporo 001-0020, Japan; 6 North-Western Provincial Health Office, Ministry of Health, Solwezi 10101, Zambia; misheckshawa@czc.hokudai.ac.jp; 7 Division of Infection and Immunity, Hokkaido University International Institute for Zoonosis Control, Sapporo 001-0020, Japan; 8 National TB Control Program, Ministry of Health, Lusaka 10101, Zambia; patrick.lungu@moh.gov.zm; 9 International Collaboration Unit, Hokkaido University International Institute for Zoonosis Control, Sapporo 001-0020, Japan

**Keywords:** *Mycobacterium tuberculosis*, CAS-Kili, recent transmission, multidrug resistance, whole-genome sequencing

## Abstract

Globally, tuberculosis (TB) is a major cause of death due to antimicrobial resistance. *Mycobacterium tuberculosis* CAS1-Kili strains that belong to lineage 3 (Central Asian Strain, CAS) were previously implicated in the spread of multidrug-resistant (MDR)-TB in Lusaka, Zambia. Thus, we investigated recent transmission of those strains by whole-genome sequencing (WGS) with Illumina MiSeq platform. Twelve MDR CAS1-Kili isolates clustered by traditional methods (MIRU-VNTR and spoligotyping) were used. A total of 92% (11/12) of isolates belonged to a cluster (≤12 SNPs) while 50% (6/12) were involved in recent transmission events, as they differed by ≤5 SNPs. All the isolates had KatG Ser315Thr (isoniazid resistance), EmbB Met306 substitutions (ethambutol resistance) and several kinds of *rpoB* mutations (rifampicin resistance). WGS also revealed compensatory mutations including a novel deletion in *embA* regulatory region (−35A > del). Several strains shared the same combinations of drug-resistance-associated mutations indicating transmission of MDR strains. Zambian strains belonged to the same clade as Tanzanian, Malawian and European strains, although most of those were pan-drug-susceptible. Hence, complimentary use of WGS to traditional epidemiological methods provides an in-depth insight on transmission and drug resistance patterns which can guide targeted control measures to stop the spread of MDR-TB.

## 1. Introduction

Worldwide, close to 4000 lives in a day are lost due to tuberculosis (TB). It is a major cause of death ascribed to antimicrobial resistance [1]. In Zambia, like many other developing countries, TB is the leading cause of death especially among people living with HIV/AIDS [2].

*Mycobacterium tuberculosis*, the cause of TB, has seven major human-adapted lineages. One of these is lineage 3 (L3) which is predominant in East Africa, the Middle East and South Asia [3,4]. Central Asian Strain 1-Kili (CAS1-Kili) forms part of lineage 3 sub-clades and is more prevalent in the eastern part of Africa [3,5].

Molecular epidemiological methods such as mycobacterial interspersed repetitive units-variable number of tandem repeats (MIRU-VNTR [6], spoligotyping [7] and IS*6110*-based restriction fragment–length polymorphism (RFLP) [8] have been applied to understand *M. tuberculosis* transmission based on genotypic clustering [9,10,11]. Nevertheless, these genotypic methods, though targeting polymorphic sites, only interrogate one percent of the *M. tuberculosis* genome and have limited discriminatory power [12,13,14]. Therefore, whole-genome sequencing (WGS) provides an ultimate method for high resolution of strain relatedness and investigation of recent transmission [15]. Further, it explores more drug-resistant mutations that occur outside the targeted regions by PCR-based assays [16]. Recently, WGS has become affordable, though it is still a very big challenge for developing nations. Based on WGS results, though not yet standardized, many reports have described strains belonging to a cluster and been involved in a recent transmission event when they differ by twelve or fewer single nucleotide polymorphisms (SNPs) and five or fewer SNPs, respectively, in their core genomes [15,17].

Previously, we reported a relatively higher percentage of lineage 3 Spoligotype International Type (SIT) 21/CAS1-Kili strains among multidrug-resistant (MDR) *M. tuberculosis* strains in Zambia when compared to other southern African countries [18]. Despite its low proportions in the region compared to predominant lineage 4, lineage 3 strains (SIT21/CAS1-Kili) were associated with MDR-TB and implicated for its spread in Lusaka, Zambia [18,19]. Among the studied MDR *M. tuberculosis* strains, 24-loci MIRU-VNTR and spoligotyping showed clonal expansion of the SIT21/CAS1-Kili strains [18]. The findings in those studies supported the possibility of recent transmission, though this could not be concluded due to the limitations of the genotyping methods used in discriminating between closely related strains. Thus, this study aims to investigate recent transmission of the MDR *M. tuberculosis* SIT21/CAS1-Kili strains in Lusaka, Zambia, by WGS.

## 2. Results

### 2.1. Cluster Analysis

Among our Zambian strains, 11/12 (92%) differed by not more than 12 SNPs, while 6 (50%) strains differed with at least one other strain by 5 or fewer SNPs. Strain 7 was closely related to strains 4, 5, and 11. Additionally, strain 7 had the lowest average SNP difference of 9 SNPs to other strains, as shown in Table 1. On the contrary, strain 6 had the highest average SNP difference of 23 SNPs to the other strains.

### 2.2. Resistance Patterns and Phylogeny

**Figure 1:**
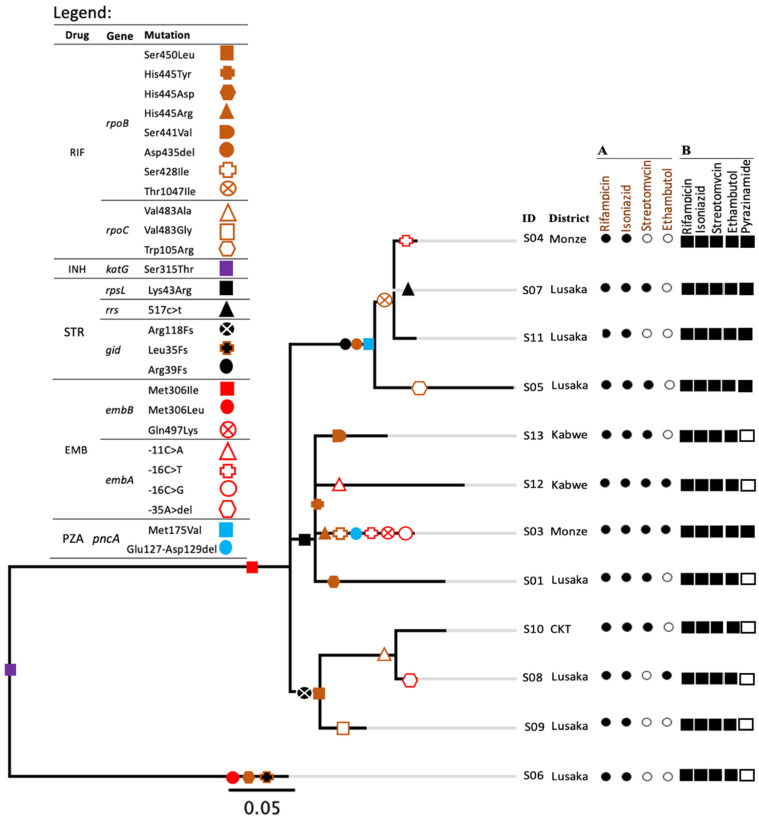
Phylogenetic tree illustrating the relations of the studied SIT21/CAS1-Kili strains. Drug-resistance-associated mutations of each strain are represented by different shapes on the branches and highlighted as indicated by the legend. Fs in the legend stand for frameshift. A, is for phenotypic drug susceptibility patterns. Black and white circles represent results of drug-resistant and susceptible phenotypes, respectively. B, Represents drug-resistant-associated mutations. Black and white squares indicate the presence and absence of drug-resistance-associated mutations to a particular drug, respectively. CTK in the district column stands for a district Chikankata. An ‘S’ letter before each ID number stands for strain. In the legend, RIF, INH, STR, EMB and PZA stand for rifampicin, isoniazid, streptomycin, ethambutol and pyrazinamide, respectively.

All strains had the same mutations for isoniazid resistance (KatG, Ser315Thr). With the exception of one strain with EmbB Met306Leu (Strain 6, Figure 1), 92% (11/12) had the same mutation associated with ethambutol resistance (Met 306 Ile) though only 25% (3/12) had the corresponding phenotypic resistance, as shown in Figure 1. Among 11 EmbB Met306Ile mutants, 36% (4/11) strains had additional mutations in *embA,* and three of these were phenotypically resistant to ethambutol. Additionally, all the 12 strains had resistance-associated mutations to streptomycin. Of these twelve strains, four and one had mutations in *rpsL* and *rrs* genes, respectively, and all were resistant to streptomycin, while 7/12 had mutations in *gid* and only two out of those seven were resistant to streptomycin. Overall, strains had different mutations towards rifampicin resistance while all RpoB Ser450Leu mutants had compensatory mutations in RpoC Val483 (Figure 1). Among four strains having RpoB Asp435 deletion, three had a mutation in RpoB Thr1047Ile and one had that in RpoC Trp105Arg. The strains seemed to have accumulated drug-resistance-associated mutations in a stepwise pattern (Figure 1). One strain (strain 3) had both −16 C > T and −16 C > G nucleotide variants in the *embA* gene. Strains having the same mutations for drug resistance to several drugs had a correspondingly small number of SNP differences between them (Table 1, Figure 1, and Supplementary Figure S1).

### 2.3. Phylogenetic Assessment of Global Sub-Lineage SIT21/CAS1-Kili (L3.1.1)

**Figure 2:**
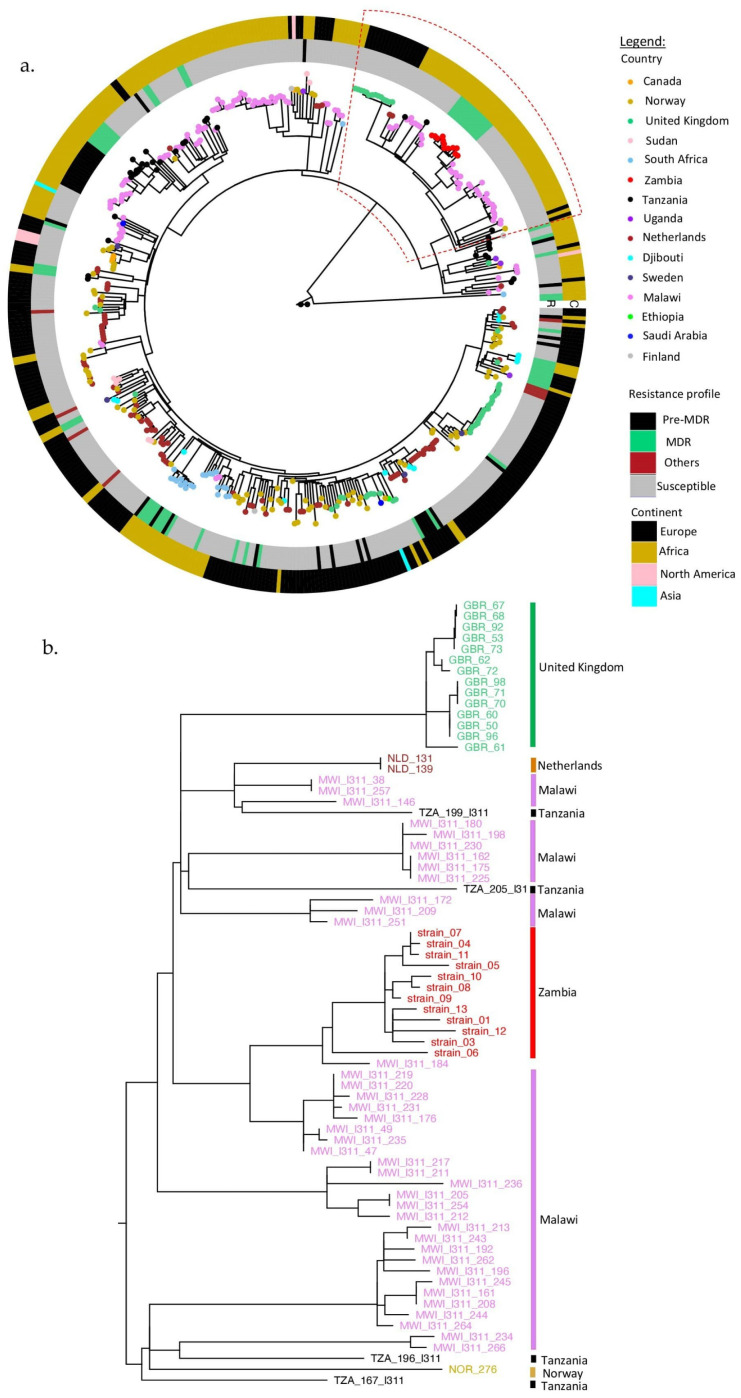
(**a**) Phylogenetic tree for global L3.1.1 strains. The countries are represented by small circles on the tips of the tree and colored as shown in the legend. The inner (R) and outer circles (C) were colored according to the drug resistance patterns and regions of strain isolation (as continents), respectively. (**b**) Enlargement of the clade containing the Zambian strains.

Most of the lineage 3.1.1 (SIT21/CAS1-Kili) strains were from Europe, 52% (250/480), followed by Africa, 48% (228/480) (Supplementary Table S1). Zambian strains formed a monophyletic clade, closer to Malawian strains but both descending from the Tanzanian strains (Figure 2). The clade having the Zambian strains also had strains from the United Kingdom (Figure 2b). The majority of the global strains were pan-susceptible (78%, 375/480), while 13% (60/480) were MDR and 2% (9/480) were resistant to other anti-tuberculosis drugs. Moreover, Zambian strains had the lowest median SNP distance when compared to strains from other countries (*p* < 0.000) (Supplementary Figure S2).

## 3. Discussion

The SIT21/CAS1-Kili *M. tuberculosis* strains were previously shown to be associated with the clonal spread of MDR-TB in the Lusaka District [18,19]. In this study, we applied WGS to investigate recent transmission events among SIT21/CAS1-Kili strains that was suggested by the combination of 24-MIRU-VNTR loci and spoligotyping.

Of the 12 evaluated SIT21/CAS1-Kili strains, 11 belonged to a cluster, as they differed by 12 or fewer single nucleotide polymorphisms (SNPs) in their core genomes, while 6 strains were involved in recent transmission events as they differed by ≤5 SNPs to at least one other strain. Therefore, WGS revealed that the SIT21/CAS1-Kili strains were closely related as they exhibited comparatively low variabilities in SNPs. In addition, several strains shared the same combinations of drug-resistance-associated mutations to isoniazid, ethambutol, rifampicin, and other anti-TB drugs. This provided more evidence on the clonal spread of MDR SIT21/CAS1-Kili strains in this region, calling for interventions to stop this possible outbreak.

Despite some reports indicating the reduced fitness as a result of drug resistance evolution [20,21], others have indicated emanating high fitness strains with preserved ability to spread [22,23]. Thus, the latter might explain the scenario in our current study. In addition, 75% (9/12) of strains had additional mutations besides the major resistance-associated mutations to rifampicin in *rpoB*, of which 33% (4/12) of strains with RpoB Ser450Leu mutation also had compensatory mutations in RpoC, Val483Ala (2/4) and Val483Gly (2/4). These compensatory mutations have been implicated in mitigating the fitness defects caused by RpoB Ser450Leu substitution in *M. tuberculosis* [24] which in turn contribute to the successful spread of MDR-TB. Interestingly, other strains had novel mutations in *rpoB* (3/12, Thr1074Ile) and *rpoC* (1/12, Trp105Arg) which were suspected to be compensatory as they occurred in association with a known mutation, Asp435 deletion (4/12), in the *rpoB* rifampicin resistance determining region (RRDR). However, this can only be confirmed by allelic exchange experiments.

Similarly, 33% (4/12) of strains had mutations in more than a single resistance-conferring gene to ethambutol. The phenomenon has been reported to be associated with high resistance to ethambutol [25,26,27,28]. In fact, all three strains which were phenotypically resistant to ethambutol in this study had multiple mutations towards ethambutol resistance. In addition, one of the three EMB-resistant strains (strain 8) had a novel mutation in the *embA* gene regulatory region (−35 A > deletion), whilst one other strain (strain 3) had −16 C > T and −16 C > G nucleotide variants in *embA*, which was suggestive of ongoing evolution within the strain. All the 12 MDR strains had mutations in codon *embB*306 which has been associated with isoniazid resistance [29], MDR, and more likely to evolve to extensively drug-resistant TB (XDR) [27,28,30]. Further, codon *embB*306 mutations have been reported to reduce susceptibility of *M. tuberculosis* to other drugs used in the treatment regimen [30,31]. Hence, reports have suggested mutations in *embB*306 as a marker for broader drug resistance than ethambutol resistance [31]. Therefore, based on this understanding, it can be speculated that possibly the strains first acquired drug resistance to isoniazid (KatG, Ser315Thr) and ethambutol (*EmbB*306) then independently became resistant to other drugs, rifampicin, streptomycin, and pyrazinamide, while propagating to the next patients (Figure 1). As a result, rapid and low-cost diagnostic techniques for detecting *embB*306 mutations in drug-resistant strains, particularly in high-burden drug-resistant TB areas, are encouraged to guide patient management and control of drug-resistant TB [28,31,32].

Even though some strains were resistant to all the first-line anti-TB drugs and streptomycin, none had resistance mutations to other second-line drugs. This at least left some treatment options for these patients, though on the other hand, calls for improved resistance detection and individualized treatment. The observed discrepancy in phenotypic and genotypic drug susceptibility patterns for ethambutol can be attributed to lack of consistency of phenotypic DST to ethambutol resistance determination as indicated by several reports [23,30]. Mutations in *embB*306, which is associated with the majority of drug-resistant strains to ethambutol, have also been detected in susceptible strains [31], whilst the same discrepancy for streptomycin might be due to low-level resistance-conferring mutations in *gid* to the drug, which may result in susceptible phenotype [33]. Mutations in *rrs* and *rpsL* genes have been associated with intermediate to high [34] and high levels of resistance to streptomycin [33,34,35,36], respectively. In addition, drug susceptibility testing is not routinely performed on all patients with TB, hence the treatment regimen in some cases may not be appropriate and this facilitates resistance amplification and further transmission of primary drug-resistant TB [32].

Phylogeny showed that some strains involved in recent transmission events were from patients residing in different districts (Monze, Chikankata) and Lusaka City (Figure 1 and Supplementary Figure S3). This conceivably suggests that there could be many unknown related intermediary cases not captured by this study hence posing a threat for more outbreaks in the future. Therefore, this calls for more improved contact tracing strategies to curtail this transmission.

Further, some previously identical strains by MIRU-VNTR and spoligotyping were delineated as unique strains by WGS, indicating its supremacy to traditional genotyping methods [37,38]. Though the application of WGS on TB epidemiology is still challenging in developing nations like Zambia, its combination where possible, with traditional genotyping methods, can make it cost-effective and be utilized as a supporting tool, especially for large-size population studies.

Generally, the global SIT21/CAS1-Kili (L3.1.1) strains were pan-susceptible to anti-TB drugs (Figure 2a). Therefore, the outbreak of drug resistance among Zambian strains might be due to local factors such as late diagnosis, poor compliance, incomplete contact investigations, or other unknown reasons in the TB control system. Interestingly, the relationship of our Zambian strains to those from Malawi and Tanzania was suggestive of the possible origin of this strain to be Tanzania, in agreement with Chihota et al., 2018 [3]. Then, it spread to Malawi before Zambia. This finding also illustrated that the TB structure in a country is likely to be influenced by TB events in the neighboring countries in addition to local factors. Therefore, regional coordination in TB control is cardinal as movements of people for trade, migration, and refuge facilitate the spread of TB [39,40]. SIT21/CAS1-Kili (L3.1.1) strains related to the Zambian and other African countries are also causing TB disease in European countries such as the United Kingdom, as suggested by the existence of these strains in the same clade. This spread can be attributed to the movements of people from the eastern part of Africa to Europe for economic activities and refuge [38,41].

The study limitations included small sample size due to poor quality DNA for some samples and inadequate patient history to precisely infer the direction for person-to-person transmission. In addition, a larger sample size with a longer collection period of the strains would have facilitated the determination of the emergency of MDR-TB and its hotspots in Lusaka. Additionally, the sample size also affected the generalization of these results. Therefore, we recommend MDR-TB surveillance on the large scale, whenever possible, to provide more information on the spread of this strain and other genotypes in the community, which can inform policy.

## 4. Materials and Methods

### 4.1. Study Samples

We used strains from the previous study conducted in Lusaka from 2013 to 2017 [18]. In the previous study, 87 MDR-TB strains were typed by spoligotyping and 24-loci MIRU-VNTR, of which 25 CAS1-Kili strains formed the largest clonal cluster and were suspected to represent a recent transmission event [18]. All CAS1-Kili strains were considered for WGS though only 13 were successfully sequenced. The other 12 strains had poor DNA quality, hence they were excluded from the study.

### 4.2. Culturing and Drug Susceptibility Testing

Briefly, sub-culturing of the isolates was performed in BACTEC™ 960 MGIT™ (Mycobacteria Growth Indicator Tube) system (Becton, Dickinson and Company, Franklin Lakes, NJ, USA) following the manufacturer’s instructions. The critical concentrations for isoniazid, rifampicin, streptomycin, and ethambutol, as prescribed by the kit manufacturer (Becton, Dickinson and Company), of 0.1, 1.0, 1.0, and 5.0 g/mL were used, respectively.

### 4.3. DNA Extraction and Genotyping

DNA was extracted by heating method as described previously [18,19]. The extracted DNA was transported to the Hokkaido University International Institute for Zoonosis Control, Japan for analysis. Further, 24-loci MIRU-VNTR [6] and spoligotyping [7] were performed as previously described [18,19].

### 4.4. Whole-Genome Sequencing Analysis

Library preparations were performed following Nextera XT DNA Library Preparation Kit (Illumina Inc., San Diego, CA, USA) manufacturer’s instructions, and Illumina MiSeq 2500 platform was used for sequencing.

WGS data analysis was carried out as previously described [42,43]. The raw paired reads were checked for quality using fastqc, results compiled using multiQC [44]. Trimmomatic was used to trim adapters, low-quality bases and filtering for a minimum read length of 20 (SLIDINGWINDOW:4:20 MINLEN:20) [45]. Variants were called using Snippy pipeline [46]; briefly, reads were aligned to the reference strain H37Rv (NC_000962.3) by Burrows Wheel Aligner (BWA), manipulated with SAMtools, and variant calling with freebayes, while variant annotation was performed with SnpEff. Further, Snippy-core generated full and core genome alignments. Gubbins [47] was further used to generate filtered polymorphic sites. Therefore, filtered SNPs in variable and invariable sites were used to calculate a pairwise distance matrix and maximum likelihood phylogenetic tree using RAxMLv8.2.11 in Geneious v 10.2.6, Biomatters, Ltd., Auckland, New Zealand https://www.geneious.com (accessed on 16 December 2021). The mean coverages of the analyzed reads for 12 CAS1-Kili strains ranged from 35.73 to 139.32 (Supplementary Table S2). One strain (strain 02) was excluded due to poor coverage. Resistance patterns and sub-lineages were determined by TB Profiler [48,49] and PhyResSE [50]. We further manually confirmed the variants using CLC Genomics workbench 10 (QIAGEN, Hilden, Germany). To understand the likely pattern of drug-resistant mutations acquisition by the strains, the drug-resistant mutations were conveniently plotted on the phylogenetic tree.

### 4.5. Phylogenetic Assessment of Global Sub-Lineage SIT21/CAS1-Kili (L3.1.1)

Sub-lineage 3.1.1 strain’s accession numbers were obtained from TB-Profiler, projects PRJEB29435 [51] and PRJEB33896 [32]. A total of 527 pairs of raw reads were downloaded from the ENA browser and 480 of these were successfully analyzed. All raw reads were processed as described above. Additionally, to avoid false hits in the repetitive regions, SNPs in PE/PPE gene families were filtered by *M. tuberculosis* BED mask file included in Snippy-package. Further, Gubbins generated filtered polymorphic sites and a maximum likelihood phylogenetic tree rooted to *M. canetti*. The phylogenetic tree was trimmed of *M. canetti* but topology maintained, for better visualization using ggtree [52,53,54,55]. To confirm the sub-lineage and drug resistance patterns for some strains, TB-Profiler was used. Further, SNP distances using Ape package [56] and Wilcoxon signed-rank test were calculated in R-studio [55]. Only countries with more than 10 strains were considered for SNP distance calculation.

## 5. Conclusions

The study revealed ongoing transmission of MDR SIT21/CAS1-Kili strains in Lusaka, Zambia, which is a public health concern and needs more evaluation. It also showed the high resolution of WGS in delineating closely related strains and determining antibiotic resistance. Further, the study supports the complimentary use of WGS with traditional methods. Intensified case finding, improved drug resistance detection and adherence to treatment can help interrupt the transmission chains.

## Figures and Tables

**Table 1:** Similarity matrix showing core-SNP differences of the 12 SIT21/CAS1-Kili strains. SNPs differences between strains ranged from 1 to 26 SNPs.

Strain ID													SNP Difference ^1^		
	S01	S03	S13	S08	S10	S09	S12	S04	S07	S11	S05	S06	≤5	≤12	≥13
S01		9	8	11	12	9	11	11	10	11	14	24	0	9	2
S03	9		7	10	11	8	10	10	9	10	13	23	0	9	2
S13	8	7		9	10	7	9	9	8	9	12	22	0	10	1
S08	11	10	9		3	5	12	10	9	10	13	23	2	9	2
S10	12	11	10	3		6	13	11	10	11	14	24	1	8	3
S09	9	8	7	5	6		10	8	7	8	11	21	1	10	1
S12	11	10	9	12	13	10		12	11	12	15	25	0	8	3
S04	11	10	9	10	11	8	12		1	2	7	23	2	8	1
S07	10	9	8	9	10	7	11	1		1	6	22	2	10	1
S11	11	10	9	10	11	8	12	2	1		7	23	2	10	1
S05	14	13	12	13	14	11	15	7	6	7		26	0	5	6
S06	24	23	22	23	24	21	25	23	22	23	26		0	0	11

Legend: 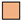 ≤ 5 SNPs; 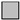 ≤ 12 SNPs; 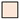 ≥ 13 SNPs. ^1^ Number of strains differing by ≤5, ≤12, and ≥13 SNPs to an individual strain. The letter S before the ID stands for strain. Boxes are highlighted according to the SNP differences as shown in the legend.

## Data Availability

Sequencing raw reads generated in this study have been deposited to NCBI, BioProject: PRJNA787003. The utilized publicly available data also analyzed in this study can be found on https://tbdr.lshtm.ac.uk/ (accessed on 12 June 2021).

## Supplementary Materials

The following are available online at https://www.mdpi.com/article/10.3390/antibiotics11010029/s1, Supplementary Figure S1: Nucleotide changes in drug-resistance-associated genes and number of strains having the same nucleotide change among the 12 SIT21/CAS1-Kili strains. The strain IDs are arranged in relation to Figure 1 (starting from the top). Fs in the table represents frameshift. Supplementary Figure S2: Comparison of median SNP distances of global representative L3.1.1 strains by country to Zambian strains. The differences in median SNP distance were significant (*p* < 0.000). Only countries with more than 10 strains were considered. Supplementary Figure S3: Map showing the residence districts of the 12 patients highlighted in different colors. Seven patients were from Lusaka, and one from Chikankata. Monze and Kabwe had two patients each. Supplementary Table S1: Number of L3.1.1 global strains from each country that were successfully analyzed. Supplementary Table S2: Information on the 12 CAS1-Kili Zambian strains.
