# Supplementary material for: Whole-Genome Sequencing Reveals Recent Transmission of Multidrug-Resistant Mycobacterium tuberculosis CAS1-Kili Strains in Lusaka, Zambia
Source: Antibiotics (Basel). 2021 Dec 28;11(1):29. doi: 10.3390/antibiotics11010029 (PMC8773284; doi:10.3390/antibiotics11010029)
Supplement: Supplementary file 1 [file antibiotics-11-00029-s001.zip › antibiotics-1495008-supplementary.pdf]

antibiotics

MDPI

Supplementary Materials

Supplementary Figure S1: Nucleotide changes in drug-resistance-associated genes and number of strains having the same nucleotide change among the 12 SIT21/CAS1-Kili strains. The strain IDs are arranged in relation to Figure 1 (starting from the top). Fs in the table represents frameshift.

Drug	Gene	Mutation	Nucleotide change	Number of strains	Strain ID											
					S04	S07	S11	S05	S13	S12	S03	S01	S10	S08	S09	S06
Rifampicin	*rpoB*	Ser450Leu	TCG > TTG	4									■	■	■	
		His445Tyr	CAC > TAC	2					■	■						
		His445Asp	CAC > GAC	2								■				■
		His445Arg	CAC > CGC	1							■					
		Ser441Val	TCG > GTG	1					■							
		Ser428Ile	AGC > ATC	1							■					
		Asp435del	GAC > ---	4	■	■	■	■								
		Thr1047Ile	ACC > ATC	3	■	■	■									
	rpoC	Val483Ala	GTG > GCG	2									■	■		
		Val483Gly	GTG > GGG	2											■	
		Trp105Arg	TGG > CGG	1				■								
Isoniazid	*katG*	Ser315Thr	AGC > ACC	13	■	■	■	■	■	■	■	■	■	■	■	■
Streptomycin	*rpsL*	Lys43Arg	AAG > AGG	4					■	■	■	■				
	rrs	517c>t	C > T	1		■										
	gid	Arg118Fs	351del	3									■	■	■	
		Leu35Fs	102del	1												■
		Arg39Fs	115del	4	■	■	■	■								
Ethambutol	*embB*	Met306Ile	ATG > ATA	11	■	■	■	■	■	■	■	■	■	■	■	
		Met306Leu	ATG > TTG	1												■
		Gln497Lys	CAG > AAG	1							■					
	embA	-11C>A	C > A	1						■						
		-35A>del	A > del	1										■		
		-16C>T	C > T	2	■						■					
		-16C>G	C > G	1							■					
Pyrazinamide	*pncA*	Met175Val	ATG > GTG	4	■	■	■	■								
		Glu127-Asp129del	380 - 388del	1							■					

Supplementary Figure S2: Comparison of median SNP distances of global representative L3.1.1 strains by country to Zambian strains. The differences in median SNP distance were significant ($p < 0.000$). Only countries with more than 10 strains were considered.

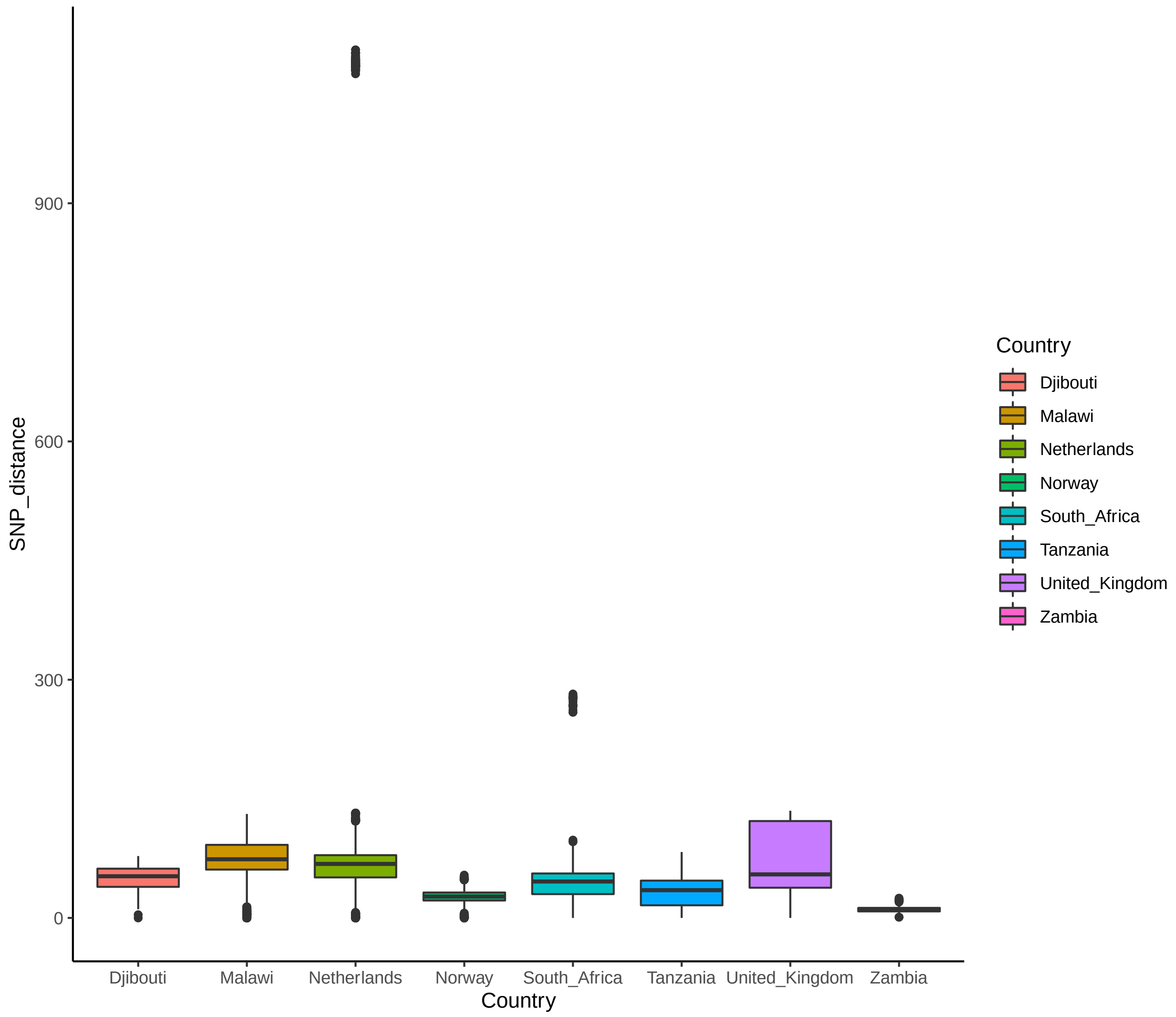

Supplementary Figure S3. Map showing the residence districts of the 12 patients highlighted in different colors. Seven patients were from Lusaka, and one from Chikankata. Monze and Kabwe had two patients each.

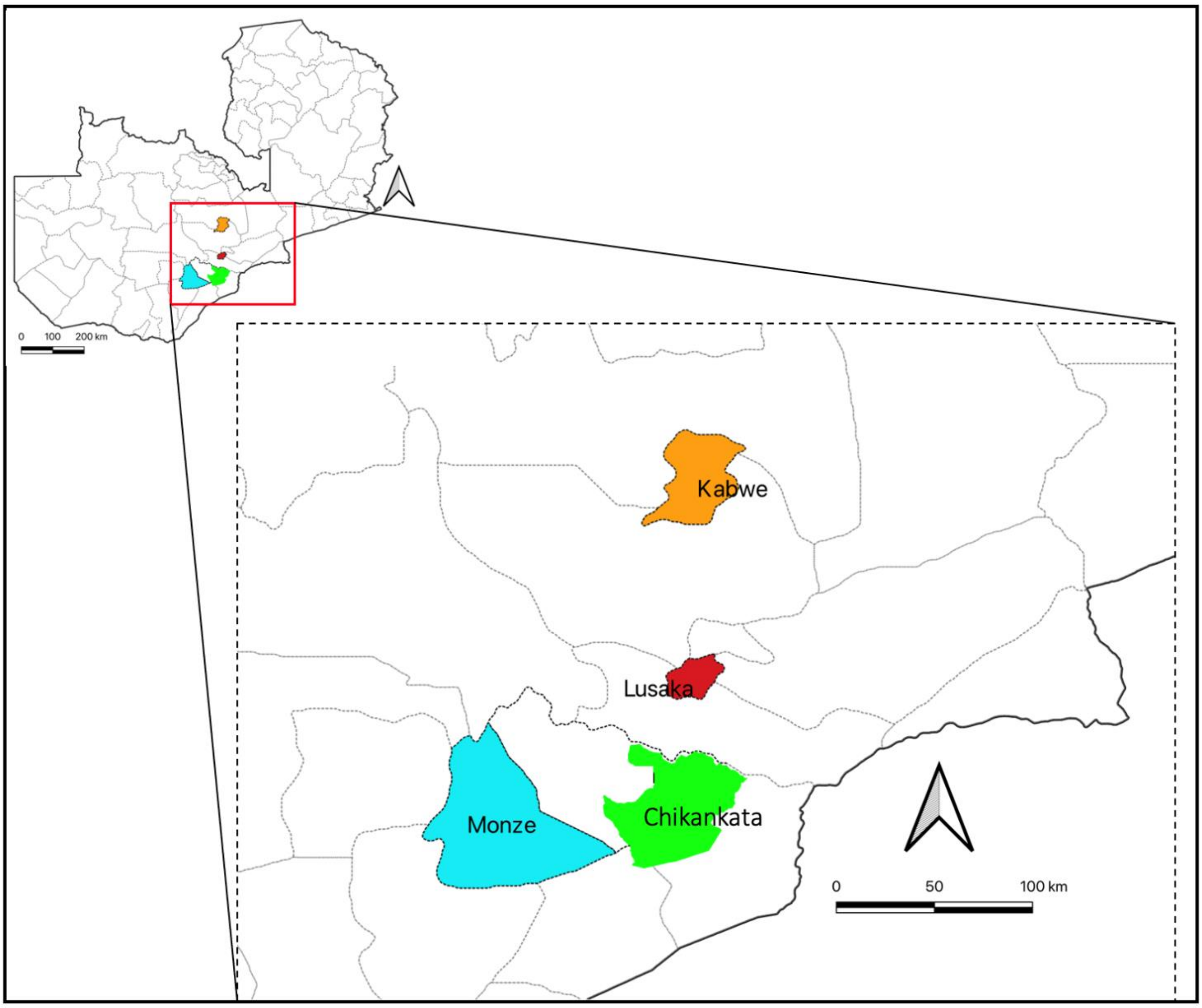

Supplementary Table S1: Number of L3.1.1 global strains from each country that were successfully analyzed.

Country	Susceptible	Pre-MDR	MDR	Others	Total
Canada	5				5
United Kingdom	48	1	1	1	51
Finland	3		1		4
Norway	69	8	3	6	86
Netherlands	88	5	6		99
Sweden	1		4		5
Saudi Arabia	2				2
Djibouti	5	2	5		12
Uganda	1	1	2		4
Malawi	119	15			134
Tanzania	16	3	17		36
Zambia			12		12
South Africa	12	1	9	1	23
Sudan	6			1	7
**Total**	**375**	**36**	**60**	**9**	**480**

Supplementary Table S2: Information on the 12 CAS1-Kili Zambian strains.

**Sample Number**	**Mean Coverage**	**Standard deviation**	**Mean read length**	**Total sequences**	**Number of reads**
S1	63.75	± 17.34	249.97	579110	1,224,370
S3	139.32	± 39.43	209.31	1522426	3,195,791
S4	35.73	± 21.4	260.14	318802	750,634
S5	118.74	± 36.57	178.23	1534056	3,292,956
S6	46.17	± 15.43	250.57	426310	900,458
S7	111.68	± 27.18	167.49	1550554	3,334,987
S8	43.29	± 13.69	254.62	387347	799,903
S9	44.75	16.08	250.83	415133	868,824
S10	73.28	± 20.76	278.31	604304	1,252,391
S11	75.05	± 21.14	266.89	641298	1,345,606
S12	76.3	± 21.52	271.42	644606	1,347,590
S13	99.68	± 25.76	212.6	1066338	2,159,212
